# Current Strategies for Promoting the Large-scale Production of Exosomes

**DOI:** 10.2174/1570159X21666230216095938

**Published:** 2023-07-10

**Authors:** Qing Qu, Bin Fu, Yong Long, Zi-Yu Liu, Xiao-Hong Tian

**Affiliations:** 1 Department of Tissue Engineering, School of Intelligent Medicine, China Medical University, 77 Puhe Avenue, Shenbei New District, Shenyang, 110122, China

**Keywords:** Exosomes, exosome production, biogenesis, exosome yield, scale-up production, clinical application

## Abstract

Exosomes, as nanoscale biological vesicles, have been shown to have great potential for biomedical applications. However, the low yield of exosomes limits their application. In this review, we focus on methods to increase exosome yield. Two main strategies are used to increase exosome production, one is based on genetic manipulation of the exosome biogenesis and release pathway, and the other is by pretreating parent cells, changing the culture method or adding different components to the medium. By applying these strategies, exosomes can be produced on a large scale to facilitate their practical application in the clinic.

## 1 INTRODUCTION

Extracellular vesicles (EVs) are nanosized, membrane-bound vesicles released from cells [1]. According to the pathway of biosynthesis or release of EVs, there are three main subtypes, namely microvesicles (MVs), exosomes and apoptotic vesicles [1]. Exosomes are about 30-150 nm in diameter and originate from endosomes formed by inward outgrowth of the plasma membrane [2]; MVs are about 100-1000 nm in diameter and arise from direct outgrowth of the plasma membrane; apoptotic vesicles are about 50 nm-2 μm in diameter and produced by apoptotic cells [3]. The International Society for Extracellular Vesicles (ISEV) recommends EVs as a generic term to describe all nanoparticles released by lipid bilayer cells. Most of the contents obtained by existing exosome isolation techniques are exosomes, but there are still other subtypes of EVs, such as MVs. Reliable exosome isolation methods and comprehensive characterization are required to describe the term exosome. For characterization, ISEV recommends using at least three protein markers with exosomal characteristics, including one transmembrane protein (CD63, CD81 or CD82), integrins, or others. In addition, a cytoplasmic protein (*e.g*., TSG101, ALIX and syntenin) and a negative protein marker (*e.g*., albumin and ribosomal proteins) are also required [4]. In addition, the size distribution and morphology of exosomes should be observed, and common methods include nanoparticle tracking analysis (NTA) and transmission electron microscopy (TEM), *etc*. Inthe rest of this review, the authors will use the term exosome when appropriate.

Almost all types of cells are capable of secreting exosomes, which may serve as a pathway for the communication of substance and signal between cells. Different cells achieve intercellular communication by secreting exosomes carrying various substances (proteins, lipids, DNA, mRNA, miRNA, lncRNA, circRNA, *etc*.), which are captured by the recipient cells for exchange [5].

**Fig. (1):**
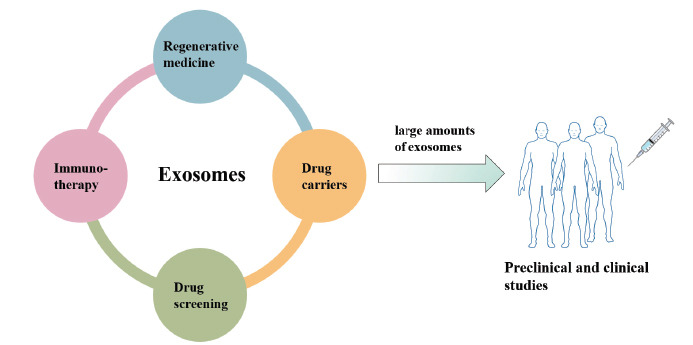
Exosome research. At present, exosomes have been used in regeneration medicine, drug carriers, drug screening, immunotherapy and other fields. However, preclinical and clinical studies require large amounts of exosomes.

Today, exosome-related research and clinical trials have penetrated many fields, such as regenerative medicine, drug carriers, drug screening, immunotherapy, *etc*. However, preclinical and clinical studies require large amounts of exosomes (Fig. **1**). In clinical trials, one patient requires approximately 100 μg/kg of exosomes per treatment [6, 7]. Therefore, meeting the need for large-scale clinical production in sufficient quantities remains a major challenge for exosome applications, which limits their therapeutic applications [8-10]. In this review, we aim to summarize different approaches to increase exosome production.

## 2 BIOGENESIS OF EXOSOMES

**Fig. (2):**
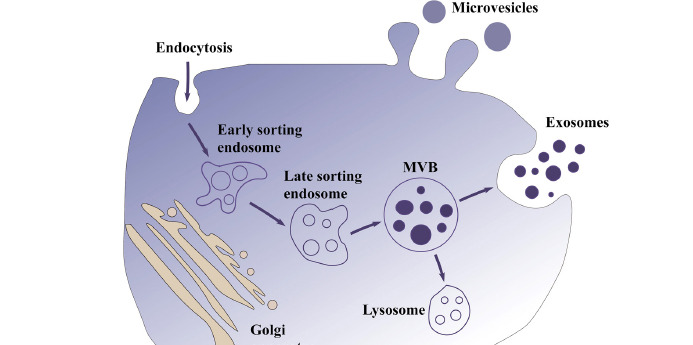
The main biogenesis pathways of exosomes. First, the cytoplasmic membrane invaginates, encapsulating some extracellular components and cell membrane proteins to form ESEs, which can exchange substances with other organelles or fuse between different ESEs to form LSEs. The LSEs further form MVBs, which will contain many ILVs. After the formation of MVBs, cells may be degraded by fusion with autophagosomes or lysosomes, or by fusion with the plasma membrane and release of substances within them.

Exosomes are initially formed by endocytosis. First, the cytoplasmic membrane invaginates, encapsulating some extracellular components and cell membrane proteins to form early endosomes (ESEs), which can exchange substances with other organelles or fuse between different ESEs to form late endosomes (LSEs). The LSEs further form multivesicular bodies (MVBs), which will contain many intraluminal vesicles (ILVs) that are formed by the inwardly recessed outgrowth of the MVBs membrane and may be released as exosomes in the future. After forming MVBs, the cells may be degraded by fusing with autophagosomes or lysosomes or by fusing with the plasma membrane and releasing the substances therein, including ILVs, which are the eventual exosomes (Fig. **2**) [11]. To release exosomes, several steps need to be completed, including the formation of ILVs in MVBs, the translocation of MVBs to the plasma membrane, and the fusion of MVBs with the plasma membrane.

The formation of MVBs synergizes through the endosomal sorting complex required for transport (ESCRT) dependent and independent pathways. ESCRT includes four complexes (ESCRT-0, -I, -II and -III) [12]. The ESCRT-0 complex recognizes and isolates ubiquitinated proteins to specific structural domains of the endosomal membrane [13, 14]. The ESCRT-I and ESCRT- II complexes are responsible for membrane bending out of bud and cargo sequestration [15]. After the formation of ILVs, the ESCRT-III complex drives vesicle scission [14, 16]. In addition, the syndecan-syntenin-ALIX pathway can also influence exosome biogenesis, which is required for the budding of CD63-positive ILVs, and is specifically controlled by ARF6 and its effector protein PLD2 [17]. A key lipid-metabolizing enzyme in the ESCRT-independent pathway is neutral sphingomyelinase (nSMase), which induces spontaneous membrane bending, invagination, and ILVs formation [18], and catalyzes sphingomyelin synthesis of ceramides that promote inward budding of MVBs. Inhibition of nSMase2 has been shown to reduce exosome release [19]. In the exosome release mechanism, SNARE proteins are able to initiate vesicle fusion. Vesicle SNARE (v-SNARE) and target SNARE (t-SNARE) form a complex to drive the fusion of the two membranes in a zipper-like manner [5]. Through this process, MVBs fuse with the plasma membrane and release exosomes into the extracellular space. In addition, Rab GTPases, HSP90, cortactin and Ca^2+^ are also involved in the exosome release process [18].

## 3 TECHNIQUES FOR IMPROVEMENT OF EXOSOME PRODUCTION

**Fig. (3):**
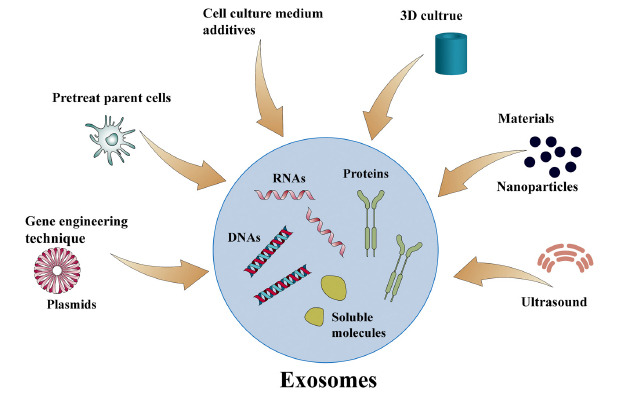
Approaches of exosome production enhancement. At present, we can increase the yield of exosomes through gene engineering technology, pretreating parent cells, 3D culture, cell culture medium additives, change of culture materials, ultrasound treatment, and other methods.

Here, we discuss the different methods used to alter exosome production, which are divided into two main strategies. One is based on genetic manipulation of the exosome biogenesis and release pathway, and the other is by pretreating parent cells, changing the culture method or adding different additives to the medium (Fig. **3**). Table **1** lists the relevant mechanisms of the methods to increase exosome yield [20-46].

### 3.1 Genetic Engineering

Genetic engineering techniques require targeting the major related genes involved in exosome biogenesis and release. Many reports have shown that genes related to exosome biogenesis (HRS, STAM1, TSG101, CHMP4, VPS4, ALIX, nSMase2, cholesterol, CD63, CD9, CD82, HSP70, *etc*.) and release (Rab family, VAMP7, VAMP8, STX5 SNAP23, *etc*.) may be disturbed by genetic engineering techniques, thus affecting cellular exosome secretion [20]. The main concern of this approach is how much the genetically engineered exosomes differ from untreated native exosomes, and further studies are needed to clarify these differences [21].

Kojima *et al.* designed an exosome-to-cell (EXOtic) device to produce large amounts of exosomes from cells, enabling efficient delivery of exosomal mRNA [22]. Plasmids for STEAP3 (associated with exosome biogenesis), syndecan-4 (enhances endosomal membrane inward outgrowth to form ILVs), and an L-aspartate oxidase fragment (accelerates the tricarboxylic acid cycle and increases cellular metabolism) are potential synthetic exosome production boosters, and co-expression of these three genes resulted in a 40-fold increase in exosome production with no change in exosome size [20, 22].

### 3.2 Pretreatment

One of the ways to influence the biogenesis and secretion of exosomes is to pretreat their parent cells. Since exosomes are produced through cellular metabolism, their composition and function are influenced by the parent cells, which can regulate their physiological functions through external stimuli.

#### 3.2.1 Hypoxic Conditions

Numerous studies have shown that mesenchymal stem cells (MSC) secrete more exosomes and enhance the therapeutic potential of exosomes in hypoxic conditions. For example, in a study of an infarcted heart model, it was found that compared to untreated MSC-derived exosomes (MSC-EXO), hypoxia-treated MSC-EXO had a better amelioration of myocardial infarction, manifested by increased vascular density, decreased myocardial apoptosis and reduced fibrosis [23]. Hypoxia can also induce vascular endothelial growth factor expression and promote angiogenesis [24].

Four transmembrane proteins (CD81, CD82, CD9) are directly involved in sorting various cargoes into exosomes, and many studies have demonstrated that four transmembrane proteins are upregulated in hypoxia [25]. For example, overexpressed CD63 and GLUT-1 are markers of hypoxic status and are associated with poor prognosis in patients with gastrointestinal mesenchymal tumors [25].

Hypoxia-inducible factor (HIF) is a family of transcription factors, and HIF proteins synthesized in normoxia are quickly degraded by Von Hippel-Lindau (VHL) E3 ligase protein. HIF can be stably expressed only in hypoxic conditions [26]. Numerous studies have shown that hypoxia enhances the therapeutic effects of MSC-EXO by increasing the expression of HIF-1α in MSC [27, 28].

In addition, the expression of HIF-1α may be positively correlated with the expression of factors related to exosome biogenesis and release. With the elevated expression of HIF-1α, the expression of ALIX, TSG101, Rab27a and Rab27b reached a peak at 12-24 h of hypoxia exposure [30]. Furthermore, Rab27a and Rab27b are key mediators of exosome release, translocating MVBs to the cell periphery and eventually fusing with the plasma membrane [47], suggesting that hypoxia-induced elevation of HIF-1α is associated with exosome biogenesis and transport of MVBs [30].

Besides HIF, other signaling molecules and pathways also play a role in exosome biogenesis in hypoxic conditions. Rab5 facilitates the formation of ESEs, and hypoxic conditions promote the aggregation of Rab5 in the perinuclear region, which potentially mediates the increased number of exosomes in prostate cancer cells [26, 29]. In addition, there is evidence that STAT3 also plays an important role in hypoxia-induced exosome production. Hypoxia can induce the activation of STAT3, which further promotes the release of exosomes in ovarian cancer cells by up-regulating Rab27a and down-regulating Rab7 (Rab7 plays a role in transporting MVBs to lysosomes for degradation [25, 31]), suggesting that hypoxia can affect the release of exosomes by blocking the degradation pathway.

The transport of MVBs also involves the binding of organelles to the cytoskeleton (actin and microtubules), related molecular motors (dynein, kinesin, and myosin), and molecular switches (small GTPases). Actin, microtubules and molecular motors are altered in different cells in hypoxic conditions [48].

Fusion of MVBs to the plasma membrane is mediated by SNARE proteins and members of the synaptotagmin family [25]. Synaptosome-associated protein 23 (SNAP-23), a type of t-SNARE, has been reported to be a substrate of pyruvate kinase M2 (PKM2), which acts as a protein kinase to phosphorylate SNAP-23 during exosome secretion, thereby promoting the fusion of MVBs with the plasma membrane [48].

In addition, the pH of the cellular microenvironment also has an effect on the biogenesis of exosomes, and acidic pH (pH = 6.0) increases the secretion of exosomes. Hypoxia within tumors is often associated with increased glycolysis and lactate accumulation in the extracellular environment, resulting in an acidic microenvironment that promotes exosome secretion [49]. It has been reported that acidic pH has been shown to alter the activation of integrins, which are key regulators of exosome uptake, so microenvironmental pH may also affect exosome entry into recipient cells [49].

#### 3.2.2 Cytokines

Nowadays, pretreatment with cytokines has become a routine to improve exosome yield and therapeutic efficacy. Compared to untreated MSC-EXO, IFN-γ-pretreated MSC-EXO showed better effects in inhibiting the proliferation of peripheral blood mononuclear cells (possibly *via* IDO), reducing pro-inflammatory cytokines, promoting *in vitro* induction of Treg, and reducing neuroinflammation and demyelination in EAE mice [50].

Multiple studies have shown that cytokines can enhance therapeutic effects by altering exosome cargoes. IL-1β pretreatment can up-regulate the expression of miR-146a in BMSC-EXO, induce macrophage differentiation into an anti-inflammatory M2 phenotype, and then play a role in ameliorating sepsis [32]. Similarly, IL-1β-pretreated MSC-EXO also exhibited significantly enhanced anti-inflammatory activity in osteoarthritis SW982 cells. This effect is mainly mediated by miRNAs, such as miR-147b, and involves the inhibition of the NF-κB pathway [33].

CD73 is widely regarded as a classic marker of MSCs [51], and its fundamental role is to mediate immunosuppression by converting ATPase to adenosine. It has been reported that the expression of CD73 is increased in gingival mesenchymal stem cells (GMSC) and their exosomes (GMSC-EXO) after TNF-α pretreatment [34]; however, the expression of CD73 mRNA in endothelial cells [52] and astrocytes [53] is not changed. It is suggested that the effect of cytokine pretreatment on CD73 may be MSC-specific, and cytokines can enhance the therapeutic effect of MSC-EXO by increasing the expression of CD73.

Promotion of the proliferation of parent cells is also one of the factors affecting exosome yield, and the effect of different concentrations of cytokines on cell proliferation varies. It is reported that low concentrations (5-10 ng/mL) of TNF-α can slightly promote GMSC proliferation, while high concentrations (100 ng/mL) of TNF-α have a slightly suppressive effect on GMSC proliferation after 48 h [54]. Therefore, the cytokine concentration needs to be balanced to achieve optimal stimulation of exosome secretion. In addition, cytokines also affect the size and morphology of exosomes. For example, TNF-α-pretreated exosomes have larger irregular shapes [55].

#### 3.2.3 Thrombin

Thrombin is a serine protease with various biological activities. Sung *et al.* [56] pretreated human umbilical cord blood mesenchymal stem cells (UCB-MSC) with different concentrations of thrombin, and the generation of EVs increased in a dose-dependent manner. The results of protein composition analysis showed that the contents of angiopoietin, angiopoietin-1, HGF, and vascular endothelial growth factor (VEGF) in EVs were increased, proving that the treatment was non-toxic and harmless to cells.

Another study showed that thrombin pretreatment enhanced the yield of MSC-derived EVs by upregulating the levels of ESEs-specific proteins, Rab5 and EEA-1, and activating the ERK1/2 and AKT pathways, and the yield of EVs increased by more than 5-fold. It is mediated by the protease-activated receptor (PAR) family, in which PAR-1 plays a central role, and PAR-3 plays an auxiliary role as an upstream mediator in this process [35].

#### 3.2.4 Adiponectin

Adiponectin is a circulating protein produced only by adipocytes, and three types of adiponectin receptors are currently known: adipoRs, calreticulin and T-cadherin [57]. Recent studies have shown that adiponectin can increase the content of ceramide in exosomes by binding to T-cadherin, thereby increasing the yield of exosomes [58]. With enhanced secretion of exosomes in the extracellular fluid and circulation, T-cadherin transmits adiponectin signaling to T-cadherin-expressing cells, such as endothelial, muscle, and cardiac cells, as well as neighboring or distant cells [57].

In addition, adiponectin and T-cadherin accumulated in MVBs increased exosome yield in cultured endothelial cells as well as in WT mouse aorta, but in T-cadherin-deficient mice, the level of exosomes in the blood was almost reduced by half, suggesting that adiponectin-induced enhancement of exosome biogenesis is dependent on T-cadherin [57]. High-molecular-weight (HMW) multimer adiponectin, a more potent form of adiponectin, increases the rate of ILVs synthesis in MVBs and significantly increases the yield of exosomes. This process is regulated by Rab27a, and after Rab27a silencing, the production of exosomes is reduced [59].

A recent proteomic study showed T-cadherin to be one of the most abundantly expressed proteins on the surface of MSC [60]. Injection of ADSC in a mouse model of ventricular hypertrophy improves left ventricular cardiac function, and studies have shown that this therapeutic effect of ADSC is largely dependent on the levels of circulating adiponectin in recipient mice, the expression of T-cadherin in ADSC and ESCRT-mediated exosome production. Knockdown of T-cadherin and ALIX (an important component of the ESCRT complex) or reduction of circulating adiponectin levels significantly attenuated the therapeutic effect of ADSC [36].

#### 3.2.5 Small Molecule Compounds

A number of studies have shown that some small-molecule compounds enhance the therapeutic potential of MSC-EXO. For example, MSC-EXO pretreated with heme, an inducer of heme oxygenase-1, can achieve cardioprotective effects by inhibiting cardiomyocyte senescence and by regulating the HMGB1/ERK pathway through miR-183-5p [61]. MSC-EXO, pretreated with a low concentration of dimethyloxaloylglycine, promoted bone regeneration and angiogenesis by activating the AKT/mTOR pathway [62]. Eugenol-treated BMSC-EVs protected tendon stem cells from damage caused by oxidative stress by activating the Nrf2/HO-1 pathway [63]. In addition, atorvastatin [64, 65], oridonin [66] and pioglitazone [67] can also enhance the therapeutic effect of MSC-EXO.

Nitric oxide (NO) is a highly reactive free radical involved in several cellular functions, including neurotransmission, regulation of blood-vessel tone, and immune response [68]. It has been shown that NO release *via* hydrogel enhances the pro-angiogenic effect of transplanted MSC [69]. The researchers found this to be due to NO promoting the secretion of MSC-EXO and enhancing the angiogenic capacity of exosomes by upregulating miR-126 expression in MSC-EXO [70].

Recently, a class of small-molecule modulators (fenoterol, norepinephrine, forskolin, N-methyldopamine and mephenesin) were shown to promote MSC-EXO secretion. Combining norepinephrine with forskolin or N-methyldopamine further enhanced exosome production by ~2.5-fold and ~3-fold, respectively [37]. These small-molecule modulators significantly upregulated the expression of MITF, nSMase2, Rab27a and Rab27b. MITF increased the expression of LSE proteins, such as Rab7 and Rab27a. nSMase2 plays an important role in ESCRT-independent pathways, and Rab family proteins are key proteins that promote exosome transport and secretion. However, these small-molecule modulators did not affect the expression of ESCRT-dependent pathway-related proteins (HRS, TSG101, STAM1, and ALIX) [37].

### 3.3 Cell Culture Medium Additives

Various experiments studying exosomes rely heavily on cell culture, but additives in culture medium, such as fetal bovine serum (FBS), contain exosomes and other macromolecular complexes. These exosomes are not only morphologically similar, but most importantly, bioactive substances in serum exosomes are taken up by experimental cells, which may interfere with the biogenesis, isolation, and downstream analysis of experimental exosomes [39]. Therefore, the removal of exosomes from FBS is of great significance for the study of exosomes [71, 72].

Today, the conventional method for removing FBS-derived exosomes/EVs is ultracentrifugation (UC) [73]. However, even up to 24 h of UC can only partially remove EVs or EV-RNA and is time-consuming and expensive. A new method based on ultrafiltration (UF) has recently been developed to remove EVs from FBS. Ultrafiltration EV-depleted FBS (UF-dFBS) was obtained by centrifuging regular FBS in Amicon ultra-15 centrifugal filters (ref: UFC910024, 100 kDa Merk Millipore Ltd., Tullagreen, Carrigtwohill, Co. Cork, Ireland) for 55 min at 3,000 g. It was compared with ultracentrifugation EV-depleted FBS (UC-dFBS) and commercial EV-depleted FBS (SBI-dFBS), and the results showed that the UF-dFBS did not contain any detectable EVs. In terms of effects on cell proliferation, the team tested the proliferation of MSC in cell culture media supplemented with UF-dFBS, UC-dFBS, and SBI-dFBS. The proliferation of cells in a cell culture medium supplemented with the three dFBS was similar at 48 h. However, cells grown in UF-dFBS and SBI-dFBS proliferated more slowly than those grown in UC-dFBS at 96 h. But these differences were not statistically significant, and all media maintained cell proliferation up to 96 h. The team showed that this may be due to the lack of large proteins and EVs. In addition, the team tested whether it was possible to increase the cell proliferation rate of MSCs grown in UF-dFBS medium. MSC cultured in UF-dFBS or UC-dFBS medium on carboxy plates proliferated significantly faster compared to normal cell-culture plates. Moreover, the increase in proliferation rate was highest for cells grown in UF-dFBS medium compared to UC-dFBS [74].

In addition to the removal of exosomes from FBS, serum-free media are increasingly used, which can alter the proliferative capacity of MSCs [75] and affect their secretory and immunomodulatory properties [76]. Studies have shown MSC-EXO cultured in a serum-free medium to exhibit better effects in terms of promoting wound healing and angiogenesis [77]. Another study reported better results in terms of sEVs yield using a new serum-free medium (Oxium^TM^ EXO) [78].

Notably, serum deprivation did not have the same effect on exosome production in different cells. Haraszti *et al.* found that although serum deprivation improved the activity of exosomes, the production of ADSC-EXO and BMSC-derived exosomes (BMSC-EXO) was significantly reduced, whereas the production of UCMSC-derived exosomes (UCMSC-EXO) was not affected, and the size of UCMSC-EXO was slightly larger than that of ADSC-EXO and BMSC-EXO [79]. In addition to quantitative changes, there were also significant differences observed in EVs’ protein composition. For example, EVs obtained from serum-free Opti-MEM medium contain higher levels of G proteins, small GTPases and kinases [38]. Ceramide plays an important role in the production of exosomes and has been implicated in the formation of ILVs within MVBs. The researchers found intracellular ceramides to be significantly upregulated and EVs to be significantly increased after culturing HEK293T with serum-free Opti-MEM medium. To confirm whether the ceramide-dependent ILVs formation pathway is the main pathway by which serum-free Opti-MEM medium promotes EVs production, the researchers observed a significant reduction in EVs after treating cells with GW4869 (nSMase inhibitor). This suggests that the majority of EVs produced in serum-free Opti-MEM medium are generated through ceramide-dependent ILVs formation [39].

However, some studies have shown potential pitfalls in the use of serum-free media. When cells reach 70-80% confluency, switching to a serum-free medium may cause unexpected cellular stress and autophagic flux, changes in the cellular phenotype, and potential alterations in EV cargo packaging and release mechanisms [80]. Therefore, EVs/exosomes extracted using a serum-free medium should be characterized in a standardized manner to increase the possibility of clinical applications.

### 3.4 Change of Culture Method

#### 3.4.1 Three-Dimensional Culture

The traditional cell culture method is the flat culture of cells, which is convenient and easy to operate. However, with the deepening of research, traditional two-dimensional (2D) culture has exhibited problems, such as low cell viability, changes in shape and morphology, and reduced proliferation and differentiation capacity. In order to solve these problems, a culture technology that can better simulate the growth environment of cells *in vivo*, that is, three-dimensional (3D) culture technology of cells, has attracted more attention.

Accumulating evidence suggests that 3D-derived exosomes (3D-EXO) are more abundant and more active than 2D-derived exosomes (2D-EXO), and can exert better therapeutic effects [81]. Macroporous or fibrous scaffolds can enhance the paracrine effects of MSC, which not only promote macrophage recruitment and enhance macrophage polarization toward a healing-promoting phenotype [82], but also enhance the capacity of MSC in tissue regeneration and repair [83]. The yield of 3D-EXO obtained by culturing cells in a hollow-fiber bioreactor was 7.5 times that of 2D-EXO, and 3D-EXO showed better activity in promoting cartilage regeneration [84]. In addition, 3D-EXO has also shown better effects in improving memory and cognitive deficits in Alzheimer's disease mice [85], protecting kidneys [86], enhancing the osteogenic capacity of BMSC [87], stimulating cell migration and proliferation, and inhibiting apoptosis [87].

The study found that the spherical structure of cells caused by 3D culture can change the microenvironment around the cells, thereby enhancing the activity and yield of exosomes. When BMSC were cultured in 3D spheroids, the central region of the spheroids was in a hypoxic state due to insufficient oxygen supply, resulting in increased exosome secretion [88].

Compared to 2D-EXO, 3D-EXO contain different protein and RNAs [89-91]. For example, the expression of F-actin was significantly reduced in 3D-cultured MSC, providing a favorable environment for the synthesis and secretion of exosomes [88]. In addition, the mRNA expression of some vesicular transport proteins (ALIX, TSG101, ADAM10, CD63, Syntenin-1, *etc*.) was also altered [40]. These changes collectively affect the production and secretion of exosomes.

#### 3.4.2 Culture Materials

Numerous studies have shown that the culture material can affect the release efficiency, speed and time of exosomes to achieve a continuous and stable collection of exosomes. For example, lithium released from biomaterials showed great potential in promoting angiogenesis, while Li-BGC-stimulated BMSC;EXO activates the PTEN/AKT signaling pathway by transferring miR-130a, thereby enhancing the pro-angiogenic capacity of endothelial cells [92]. Chitosan (CS)/β-glycerophosphate (β-GP) hydrogels have attracted much attention due to their thermosensitive properties and injectability [93]. The exosome-loaded CS/β-GP hydrogels were able to target SPRY2 *via* exosomal miR-21, significantly prolonging the delivery and release of exosomes, promoting angiogenesis, and enhancing bone regeneration [85].

Collagen, a commonly used natural biomaterial, is an important component of the extracellular matrix (ECM), which is not only a tissue support but also participates in cell migration, differentiation, and proliferation [94]. The study has integrated human placental mesenchymal stem cell-derived EVs into a collagen matrix. Due to the degradable properties of the collagen matrix, the EVs inside are slowly released into the surrounding tissues. The results showed that the collagen matrix could enhance the cytoprotective ability of EVs by inhibiting the apoptotic pathway and DNA damage, significantly enhancing the retention and stability of EVs in the ischemic kidney [95], suggesting that the collagen matrix is an effective material for enhancing the therapeutic effect of EVs.

Recently, Ji *et al.* fabricated a microgrooved substrate to stimulate rat adipose-derived mesenchymal stem cells (rASC) to generate more sEVs, which may be related to the up-regulated expression of HRS, ALIX, and Rab35 in rASC. In addition to yield, the cargo of sEVs was also altered with elevated pro-angiogenic miRNAs and growth factors, exhibiting enhanced pro-angiogenic properties [41]. Another study used 45S5 Bioglass^®^ (BG) to promote MSC-EXO production, enhanced ceramide and RabGTPase pathways, and altered cargo expression in exosomes [96].

In addition to biomaterials, nanomaterials can also significantly enhance the role of cells and exosomes in intercellular communication [97]. Park *et al.* synthesized a bioavailable nanoparticle (NPs) based on iron oxide and polylactic-co-glycolic acid or PLGA. The NPs were transported into lysosomes by lectin-mediated endocytosis, and the nanoparticles stimulated autophagy-related factor (Rab7, involved in MVBs and autophagosome formation) to release MSC-EXO [98]. In addition, nanoparticles can also promote the release of exosomes by modulating the ceramide pathway (by modulating the activity of nSMase), oxidative stress, endoplasmic reticulum stress, and apoptosis [99, 100].

### 3.5 Cell Source

The production and secretion of exosomes are subject to many regulations, among which the source of MSC has a great influence on the production and secretion of exosomes [101]. Therefore, the selection of MSC is crucial to improve the yield of exosomes. There are many sources of MSC, including adipose tissue, umbilical cord, bone marrow, placenta, *etc*. [102]. Studies have shown that amniotic fluid-derived MSCs (AFMSC) release more exosomes than BMSC [103]. Umbilical cord mesenchymal stem cells (UCMSC) grow faster (~4 days doubling time) than BMSC or ADSC (~7 days doubling time), and UCMSC-derived exosomes are not only larger than those from BMSC and ADSC but they are also produced in higher yields, with each UCMSC producing 4 times more exosomes than BMSC or ADSC [90].

In addition, the therapeutic effects of exosomes from different stem cells are also different. For example, LujunJi *et al.* found that compared with BMSC-EXO, dental pulp mesenchymal stem cell-derived exosomes (DPSC-EXO) had stronger immunomodulatory activity and they more effectively induced apoptosis of CD4^+^ T cells. However, the effect of inhibiting the proliferation of CD4^+^ T cells is similar to that of BMSC-EXO [104].

### 3.6 Others

Physical stimulation, such as ultrasound, also have a great effect on the secretion of exosomes. Low-intensity pulsed ultrasound (LIPUS) can enhance the cartilage regeneration effect of BMSC-EXO, possibly by suppressing the inflammatory response by inhibiting the NF-κB signaling pathway [105]. Yang *et al.* found that the anti-inflammatory effect of LIPUS is the result of a combination of up-regulation of anti-inflammatory genes, immunosuppressive cells, and various EV/exosome biogenesis-related genes, of which the expression of Rab11 is as high as 2.9-fold [42]. It has been reported that Ca^2+^ plays a crucial role in the transport of MVBs and fusion with the plasma membrane. Cancer cells exposed to surface-reflector body waves (high-frequency acoustic stimulation) enhanced exosome production by 8-10-fold through a calcium-dependent mechanism, along with increasing the expression of ALIX, TSG101 and CD63 [43]. However, the reproducibility and effectiveness of ultrasound are poor, so it is necessary to explore the appropriate exposure conditions of different donor cells to ultrasound and to evaluate the influence of sonicated sample properties [106].

H_2_O_2_ is a common oxidant, and exposure of cells to high concentrations of H_2_O_2_ can lead to cell damage and death, while low concentrations of H_2_O_2_ can improve cellular tolerance to oxidation. After the treatment of MSC with low concentrations of H_2_O_2_, the ability of the released exosomes to promote angiogenesis was significantly enhanced [107]. This effect may be achieved by altering the cargo in EVs/exosomes, with 10 anti-angiogenic miRNAs decreased and 2 pro-angiogenic miRNAs increased in EVs. In addition to miRNAs, the content of 17 proteins was also altered, of which 12 were involved in angiogenesis [44].

Alcohol can regulate exosome secretion by increasing the transcription of various Rab proteins, v-SNARE and t-SNARE, involved in exosome biogenesis and secretion pathways in hepatocytes [45]. In addition, alcohol dose-dependently increased the level of MMP2, a marker for assessing exosome activity, in microglia-derived exosomes [46].

### 3.7 Scale-up Production of Exosomes

Exosomes are a promising tool for regenerative therapy. In the field of regenerative medicine, most of the current researches focus on the application of MSC-EXO, but due to age constraints, MSC cannot be applied to the clinic on a large scale. Ana and colleagues reported one of the attempts to achieve MSC immortality. They designed a PiggyBac™ (PB) transposon system to simultaneously overexpress human polycomb ring finger proto-oncogene BMI1 and the human telomerase reverse transcriptase gene in MSC using a non-viral integration vector to achieve canine ADSC immortality and maintain the proliferation and differentiation potential of MSC for a longer period of time [108]. Lai *et al.* created immortalized MSC by transfecting MYC-carrying lentiviral particles, and they suggested that the use of exosomes rather than cells as therapeutic agents could reduce the risk of lentiviral integration [109]. However, the analysis of biological functions alone cannot confirm that the process of cell transformation does not affect the properties of exosomes. Therefore, further analysis is required to identify possible changes in exosome content [21].

Yang *et al.* developed a cellular nanoporation (CNP) biochip to stimulate cells to produce and release exosomes containing nucleotide sequences of interest, including mRNA, miRNA, and shRNA. Compared with other traditional electroporation methods, the yield of CNP-EVs was increased by 50 times, and they could be continuously secreted for 24 h regardless of cell source or transfection vector, and cells prepared at 37°C produced more EVs than those at 4°C. They also found that CNP significantly enhanced the formation of MVBs and ILVs, and the increase of intracellular Ca^2+^ may be the initial factor for CNP-induced exosome secretion. Furthermore, the heat shock response in the context of CNP-induced focal heat stress promoted the activation of P53-TSAP6 signaling, resulting in increased exosome production. When administered *in vivo*, therapeutic exosomes exhibited long circulating half-lives and minimal cytotoxicity. The study showed that a single CNP cycle lasting 2-3 days can generate ~10^12^ therapeutic exosomes per 1cm×1cm chip with ~10^6^ cells, which is sufficient for preclinical *in vivo* studies when multiple chips are used. In addition, larger CNP chips that can transfect more cells and microfluidic-enhanced CNP processes are currently being developed. The next step will be to evaluate multiple allogeneic and autologous cell sources to select the optimal cell source for the production of therapeutic exosomes [110].

**Fig. (4):**
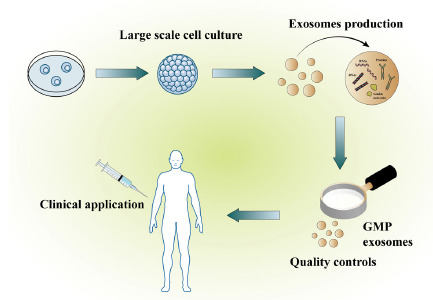
Schematic diagram of exosome production for clinical applications. Firstly, the cells were cultured on a large scale; secondly, exosomes were prepared according to the standard requirements of GMP; and finally, exosomes were applied in the clinic after strict quality inspection.

Studies have shown that 0.5 to 1.4×10^11^ exosomes are required per patient in clinical trials [111]. Clinical application of exosomes requires compliance with Good Manufacturing Practice (GMP) standards (Fig. **4**). A study has reported the possibility of tangential flow filtration (TFF) for reproducible large-scale production of GMP-grade exosomes. The productivities of ADSC-EXO from different batches ranged from 1.05×10^11^-2.36×10^11^ particles per liter of CM. Compared to ADSC-EXO isolated by ultracentrifugation, ADSC-EXO isolated by TFF may exhibit different cargo properties and different modes of disease-modifying action [112]. It has been reported that the use of human platelet lysate (HPL) instead of FBS can promote the proliferation of MSC [113]. However, the presence of high fibrinogen in HPL promotes fibrin gel formation when diluted into calcium-containing media. To circumvent these drawbacks, Karin Pachler and colleagues developed and tested a protocol for the production of pHPL-supplemented media without fibrinogen, non-human components, and pHPL-derived EVs [114]. Their results suggest that pHPLEVs-depleted medium is suitable for BMSC large-scale propagation.

Andriolo *et al.* developed a method for large-scale production of GMP-grade exosomes from cardiac progenitor cells, including complete upstream culture, downstream purification and quality control with exosome recovery rate ≥ 58%, total protein removal rate of 97-98%, and the full scale run yielding 3.1×10^13^ exosome particles. The team believes that this manufacturing method can also be applied to exosomes from other cell sources (*e.g*., MSC, muscle cells, fibroblasts, nervous system cells, dendritic cells, *etc*.) for different clinical applications [115].

Another study reported a clinical trial of large-scale production of GMP-grade engineered exosomes (iExosomes) from BMSC for pancreatic cancer patients. The authors developed a bioreactor-based system for large-scale production of MSC-EXO as well as a procedure to electroporate siRNA into exosomes using clinical-grade diluents to target genes of interest. In addition, they further tested the stability of iExosomes and found negligible loss of antitumor efficacy when stored at –80°C for up to 5 months. Notably, the exosome production used in the study was based on ultracentrifugation, which may include non-exosomal contaminants, but the results showed that their iExosomes did not produce any measurable side effects and showed consistent *in vitro* and *in vivo* efficacy [116].

## 4 ISOLATION AND PURIFICATION OF EXOSOMES

The yield and purity of exosome-based therapeutics depend not only on the production methods but also on the effectiveness of the exosome isolation method. Ultracentrifugation (UC) is the most commonly used method for exosome isolation [117]. However, UC step damages the vesicles and leads to aggregation, which can ultimately affect the downstream analysis or application of EVs [118, 119].

Due to the limitations of UC, researchers are constantly developing new methods to achieve high efficiency, purity and yield of exosome isolation. Techniques, such as size exclusion chromatography (SEC), filtration, flow field-flow fractionation (FFF), electrophoresis and dielectrophoresis (DEP), are becoming the preferred methods for efficient exosome isolation [120]. Furthermore, a combination of multiple methods is usually the best choice for exosome isolation, as the diverse, complex and heterogeneous origin of exosomes requires sophisticated isolation methods.

Visan *et al.* [121] proposed the use of a combination of TFF and SEC for sEV isolation from cells grown in medium-containing serum. sEV was isolated from HeLa cells using this method with at least 10 times more particles than UC. Moreover, in terms of processing time, UC takes 14 hours, while the latter takes less than 3 hours. In addition, the equipment for UC is very expensive, while all the materials required for TFF add up to less than one-tenth of the cost of the equipment for UC. According to the team, this method significantly improves sEV yields without affecting the particle morphology and is specific and reproducible. In addition, it minimizes the use of cell culture-related consumables, saves costs, reduces time consumption, and has scalability potential for downstream applications. However, they suggested that the increased sEV yield produced by TFF presumably compensates for the albumin contamination. Nevertheless, UC is not effective in removing albumin. For downstream applications where yield and albumin presence that do not interfere with the analysis are a priority, TFF is an optimal isolation method.

Chernyshev *et al.* [122] developed a novel asymmetric depth filtration (DF) method as a new alternative for unbiased separation of high-purity EVs. Compared to UC and SEC, EV biomarkers (CD63, CD81, CD9 and EpCAM) were most expressed in DF-isolated samples with the least contamination of protein and membrane debris. It can be scaled up by processing multiple centrifuges simultaneously until the rotor capacity is reached.

Currently, there are no “one-size-fits-all” standalone techniques for EV isolation [120]. Since different techniques and methods have both advantages and disadvantages and have different principles for isolation and separation, a combination of techniques, such as SEC with filtration-based methods, has become a common trend in numerous publications in order to obtain the most suitable EVs for the desired applications [120].

## 5 EDIBLE EXOSOMES-LIKE NANOPARTICLES

In addition to animal-derived exosome particles, plant-derived exosome particles also play an important role in the treatment of diseases. A recent interesting development is the isolation of EVs from dietary sources in the exosomal size range, which can be collectively termed edible exosomes-like nanoparticles (ELNs) [123]. ELNs are plant-derived membrane-bound particles released by microvesicular bodies for cellular communication and regulate immune responses against many pathogens. Studies have identified their role as cytoprotective agents as they carry bioactive material as cargoes transferred to recipient cells and affect various biological functions in the host. ELNs have the characteristics of small size, biocompatibility, stability, low toxicity, and non-immunogenicity, which make them better therapeutic candidates than other mammalian EVs [124].

Edible ELNs are known to be important for the treatment of many diseases related to the intestinal barrier and gut microbiota homeostasis. A study has reported that lemon exosome-like nanoparticles protect mice from Clostridioides difficile infection by enhancing the survivability of probiotics. In addition, they can increase the levels of I3Ald and I3LA, and decrease the intestinal indole level, thereby activating the expression of IL-22. It is reported that IL-22 plays a key role in reducing the severity of many intestinal infections [125]. Wang *et al.* demonstrated that grapefruit-derived ELNs enhanced the anti-inflammatory capacity of intestinal macrophages and ameliorated dextran sodium sulfate (DSS)-induced colitis in mice [126].

In addition, edible ELNs have been found to be useful for anti-inflammatory, anti-tumor activity and hepatoprotection [127]. Ginger-derived nanoparticles can reduce DSS-induced acute inflammation by decreasing the expression of proinflammatory cytokines, increasing the expression of anti-inflammatory cytokines, and limiting the production of reactive oxygen species by phagocytes. They have also been found to reduce colon tumorigenesis and growth by decreasing inflammation-induced proliferation of intestinal epithelial cells [128]. Ginger derived nanoparticles have been shown to prevent alcohol-induced liver injury by mediating the activation of nuclear factor erythroid 2-related factor 2 (Nrf2) [129]. Stimulation of Nrf2 has been reported to lead to hepatic expression of detoxification and antioxidant genes, which contribute to hepatoprotection [127]. It has been shown that mitochondria altered by oxidative stress are thought to play an important role in cell death [130]. The combined effects of multiple bioactive molecules contained in tea flower-derived exosome-like nanoparticles trigger high oxidative stress in cancer cells, resulting in mitochondrial damage, cell cycle arrest, and the subsequent cell apoptosis, which helps to inhibit metastatic breast cancer [131].

ELNs can be considered future therapeutic approaches, especially for gut and liver diseases. As technology advances, the techniques of isolation, enumeration and characterization make them easier to be used as a therapy. However, more research is still needed to take this simple, powerful and affordable treatment or drug delivery system to the next level for the benefit of patients suffering from advanced intestinal or liver diseases.

## CONCLUSION

Exosomes are currently a hot topic, but clinical applications require a large number of exosomes. From physical conditions to biomolecular regulation, researchers have proposed a variety of methods to improve the yield and quality of exosomes. These different treatment methods may have different effects in terms of enhancing the effect of exosomes, and different treatments can be selected according to the purpose of achieving the desired effect.

Notably, exosomes generated by methods that manipulate cellular origin or conditions require further characterization of their composition. These approaches may affect the biological function of exosomes, potentially altering cells fundamentally in ways we have not yet recognized and potentially introducing new and poorly defined risks to research subjects. In addition, the use of more effective isolation and purification methods is key to improving the quantity and quality of exosomes. In the future, researchers may need to integrate multiple methods to form standardized and consistent quality procedures to propose methods for the large-scale production of clinical-grade exosomes.

## Figures and Tables

**Table 1:** Methods of increasing exosome yield and quality.

**Methods**	**Cell Type**	**Suggested Mechanism of Action**
Genetic engineering	—	Introduce foreign genes into recipient cells after *in vitro* recombination [20]
Hypoxic conditions	Tumor cells	SNAP-25, PKM2 and SNAP-23↑[25]
Hypoxic conditions	Prostate cancer cells	Rab5↑ [26, 29]
Hypoxic conditions	MSC	HIF-1α, ALIX, TSG101, Rab27a and Rab27b↑[30]
Hypoxic conditions	Ovarian cancer cells	STAT3 activation, Rab27a↑, Rab7↓[31]
Cytokines	MSC	miR-146a↑[32], miR-147b↑ [33], CD73↑[34]
Thrombin	MSC	Rab5 and EEA-1↑[35]
Adiponectin	MSC	T-cadherin↑ [36]
Fenoterol, norepinephrine, trichothecene, N-methyldopamine and mephentermine	MSC	MITF, Rab27a and Rab27b↑ [37]
Serum-free Opti-MEM medium	MSC	G proteins, small GTPases and kinases↑ [38]
Serum-free Opti-MEM medium	HEK293T cells	Ceramide↑ [39]
3D culture	Human induced pluripotent stem cells	The mRNA expression of ALIX, TSG101, ADAM10, CD63, and Syntenin-1 was altered [40]
Biomaterials	MSC	HRS, ALIX and Rab35↑ [41]
Ultrasound	Not mentioned	Multiple exosome biogenesis-related genes were upregulated, with Rab11 expression up to 2.9-fold [42]
Ultrasound	U87-MG human glioblastoma cells and A549 adenocarcinomic human alveolar basal epithelial cells	Enhanced production of exosomes *via* a calcium-dependent mechanism, resulting in an 8-10-fold increase in yield. ALIX, TSG101 and CD63↑ [43]
H_2_O_2_	MSC	Altered miRNA and protein expression in EVs [44]
Alcohol	Human hepatocytes	Rab proteins, v-SNARE and t-SNARE↑[45]
Alcohol	Microglia	MMP2↑[46]

## LIST OF ABBREVIATIONS

CNP: Cellular Nanoporation
ELNs: Exosomes-like Nanoparticles
EVs: Extracellular vesicles
FFF: Field-flow Fractionation
HIF: Hypoxia-inducible Factor
HMW: High-molecular-weight
HPL: Human Platelet Lysate
ILVs: Intraluminal Vesicles
LSEs: Late Endosomes
MSC: Mesenchymal Stem Cells
MVBs: Multivesicular Bodies
NO: Nitric Oxide
NTA: Nanoparticle Tracking Analysis
PAR: Protease-activated Receptor
SEC: Size Exclusion Chromatography
TEM: Transmission Electron Microscopy
UC: Ultracentrifugation
VEGF: Vascular Endothelial Growth Factor
VHL: Von Hippel-lindau
